# An IQ consortium analysis of starting dose selection for oncology small molecule first-in-patient trials suggests an alternative NOAEL-based method can be safe while reducing time to the recommended phase 2 dose

**DOI:** 10.1007/s00280-023-04570-3

**Published:** 2023-07-28

**Authors:** Bart A. Jessen, Paul Cornwell, Sean Redmond, Thomas Visalli, Marie Lemper, Todd Bunch, Timothy Hart

**Affiliations:** 1 Pfizer, Drug Safety Research and Development, San Diego, CA 92121 USA; 2 Eli Lilly, Nonclinical Safety Assessment, Indianapolis, IN USA; 3 Clinical Pharmacology & Safety Sciences, AstraZeneca Pharmaceuticals, Waltham, MA 02451 USA; 4 Eisai Inc., Global Nonclinical Regulatory, Nutley, NJ 07110 USA; 5 Development Science, UCB, Inc., Cambridge, MA 02140 USA; 6 Nonclinical Safety Evaluation, Bristol Myers Squibb, Princeton, NJ 08540 USA; 7 GlaxoSmithKline, IVIVT, Collegeville, PA 19426 USA

**Keywords:** First-in-patient, Recommended starting dose, NOAEL-based

## Abstract

The first-in-patient (FIP) starting dose for oncology agents should be reasonably safe and provide potential therapeutic benefit to the patient. For late-stage oncology patients, this dose is often based on the ICH S9 guidance, which was developed primarily based on experience with cytotoxic chemotherapeutic agents using the rodent STD_10_ or non-rodent HNSTD and an appropriate safety factor. With the increase in molecularly targeted chemotherapeutics, it is prudent to re-evaluate how the FIP dose is derived to ensure that the appropriate balance between risk and therapeutic benefit to the patient is achieved. Blinded data on 92 small molecule oncology compounds from 12 pharmaceutical companies who are members of the IQ DruSafe consortium were gathered to investigate if a NOAEL-based starting dose without a safety factor would have been tolerated in the FIP trial and if so, estimating how many dose escalation cohorts could have been reduced. Our analysis suggests that the NOAEL-based alternative starting dose would have been tolerated in most cases evaluated, with an anticipated mean reduction of 2.3 cohorts. Of the 12 cases where the alternative approach resulted in a starting dose that would have exceeded the MTD/RP2D, none of the nonclinical toxicities in these cases were considered irreversible and would be monitorable in all but one instance. Most non-tolerated cases were within two–threefold of the MTD/RP2D, with the clinical AEs considered manageable and mitigated by dose de-escalation. No one method of FIP dose calculation will likely be appropriate for all oncology small molecules and starting dose selection should be performed using a case-by-case approach. However, the NOAEL-based method that does not utilize a safety factor should be considered when appropriate to minimize the number of patients exposed to sub-therapeutic doses of an investigational oncology agent and accelerating development to RP2D.

**Supplementary Information:**

The online version contains supplementary material available at 10.1007/s00280-023-04570-3.

## Introduction

The first-in-patient (FIP) nonclinical safety package is designed to enable an initial risk assessment of the drug candidate, identify appropriate clinical safety monitoring, and define the recommended starting dose (RSD) for the FIP clinical trial. The FIP trial for oncology agents is generally conducted in patients with advanced malignancies, which necessitates careful selection of the RSD to be sufficiently safe, while also potentially providing therapeutic benefit to late-stage cancer patients with limited therapeutic options that enrolled in these trials. Therefore, an optimal balance between safety and clinical benefit is desired, such that the number of patients in initial and subsequent cohorts exposed to potential sub-optimal therapeutic doses of the investigational agent is minimized [1].

Currently, the RSD approach for small molecule oncology agents is detailed in the International Council for Harmonisation (ICH) S9 guidance [2, 3] where the FIP start dose is based on one-tenth the severely toxic dose in 10% (STD_10_) of rodents or one-sixth the highest non-severely toxic dose (HNSTD) in non-rodents, as appropriate or sensitive, with interspecies scaling. DeGeorge et al. suggested a slightly different method, in which one-tenth STD_10_ in rodents can define the RSD as long as one-tenth STD_10_ in rodents does not cause serious irreversible toxicity in a non-rodent species [4]. If one-tenth of the rodent STD_10_ causes serious irreversible toxicities in non-rodents, then one-sixth of the non-rodent HNSTD is used to define the RSD. These approaches have largely met the development needs of chemotherapeutics with cytotoxic mechanisms. However, since the approval of ICH S9, there has been a shift in development to novel molecularly targeted agents [5]. Molecularly targeted agents (MTA) are based on different mechanisms of action, and these targeted therapies may sometimes require alternative approaches to identify appropriate dose ranges [6]. Therefore, it is prudent to re-evaluate how the RSD is derived for small molecule oncology agents to ensure we continue to optimize oncology clinical trial starting dose justification for the appropriate balance between risk and potential therapeutic benefit.

Selection of starting dose for clinical trials depends on population (healthy volunteers or patients) and mechanism of action. Prior scientific publications have reviewed the potential approaches to determine the FIP starting dose, with authors generally focusing on the previously described toxicology-based STD_10_ and HNSTD methods [1, 5]. Use of these methods are not considered appropriate for immune activating drugs for oncology indications where a minimum anticipated biological effect level (MABEL) approach is recommended, and also do not account for the potential nonclinical toxicity profile of novel MTA, when a toxicity-based approach for starting dose selection may be too conservative [5]. An assessment of a no-observed-adverse-effect level (NOAEL) defined in the in vivo toxicology studies to determine the clinical starting dose is typically used in non-cancer therapeutic indications only and includes the addition of safety factors [7].

A review of 59 approved small molecule oncology MTAs [5] showed 58 of these FIP trials had a starting dose lower than the maximum tolerated dose (MTD) in oncology patients, which indicated starting doses based on nonclinical toxicology data were safe. The majority of clinical trials used a 3 + 3 design approach, with a median of four dose escalation steps required to reach MTD or recommended Phase 2 dose (RP2D) from the starting dose. An earlier review of 81 FIP cancer trials identified from the Scopus abstract database indicated that the derivation of the starting dose was most often based on a MTD from toxicology studies and/or considering the type of nonclinical toxicity [1]. This analysis showed that starting doses for MTAs were based on rodent and non-rodent data in similar proportions. Overall, starting doses selected using such a toxicology-based method were safe in most cases and did not exceed the human MTD in 96% (*n* = 81) of Phase I clinical trials of MTAs reviewed.

Since the FIP starting dose for oncology therapeutics is now based on the ICH S9 guidance (or similar guidance from [4]), which was generally modeled based on experience with cytotoxic chemotherapeutic agents and is not always appropriate for current oncology products in clinical development, an alternative approach was evaluated. For this exercise, blinded data on 92 small molecule oncology compounds from 12 pharmaceutical companies that had completed dose escalation in cancer patients were gathered with the goal of investigating if a different method for selecting the RSD based on a species justified NOAEL without an added safety factor could be used to select a starting dose that was both tolerated and enabled a reduction in dose escalation cohorts and, thus, reducing the number of cancer patients exposed to potentially sub-therapeutic doses. This approach is consistent with recent FDA guidance on severely debilitating life-threatening hematological diseases where starting dose selection can be justified using HNSTD, STD10, or NOAEL without defining safety factors [8].

## Methods

An IQ DruSafe Working Group was formed to evaluate an alternative approach to setting starting doses in oncology FIP clinical studies. The scope was small molecule marketed MTA oncology drugs or those in clinical development that had reached an MTD/RP2D. A database was created capturing 50 fields of information for each of the 92 anonymized compounds provided by 12 pharmaceutical companies (AbbVie, AstraZeneca, Amgen, Boehringer Ingelheim, Bristol Meyers Squibb, Eisai, Eli Lilly, GlaxoSmithKline, Janssen, Merck, Novartis, and Pfizer). An additional 9 fields of data were collected for the alternative starting dose scenario exercise, for a total of 5428 data points. The information collected related to the compound attributes, and nonclinical and clinical safety data. These included general target class, nonclinical toxicology species, and toxicology or clinical study information. Nonclinical and clinical information included doses, exposures (AUC), dose-limiting target toxicities, NOAEL, STD_10_, HNSTD, MTD/RP2D, dose regimens, and number of clinical dose escalation cohorts needed to reach MTD/RP2D. Many database fields used drop-down menus with fixed responses to facilitate downstream analysis. To protect the anonymity of the company data, specific compound identifiers and molecular targets were not included. The additional 9 fields collected data for the alternative starting dose exercise using either actual study NOAELs or a study-justified NOAEL (toxicities not considered dose-limiting in cancer patients, e.g., if testes toxicity in rodent or non-rodent did not support a NOAEL determination) to calculate a NOAEL-based starting dose without an additional safety factor. The allometrically scaled NOAEL-based starting dose without a safety factor was compared to the reported MTD/RP2D to assess whether it exceeded that dose. If the alternative starting dose was below the MTD/RP2D, the number of dose escalation cohorts needed to reach the established MTD/RP2D was estimated (assuming a similar approach to dose escalation, e.g., dose doubling) and the difference between the number of actual and alternative escalation cohorts calculated. The database was curated for entries exhibiting obvious errors or lacking data necessary to meet basic criteria, corrections requested by IQ Secretariat to preserve data confidentiality, and the data exported to Microsoft Excel for analysis (Supplemental Data).

## Results

### Overview

The companies were asked to characterize the target class for the compound (Table 1). Comparing the results of the alternative assessment to the target class would assist in identifying mechanistic classes that carried higher risks for this approach.

**Table 1:** Target class

Drug target class	Number of compounds submitted (%)
Signal transduction	39 (42)
Cell cycle/DNA damage repair	12 (13)
Epigenetic	11 (12)
Immuno-oncology	11 (12)
Apoptosis inducer	9 (10)
Other*	10 (11)

*Other (*n*) = cancer mutation (4), proteosome inhibitor (3), hormone therapy (2), metabolic (1)

With regard to the MTA nonclinical toxicology FIP-enabling packages, 74% were conducted after the implementation of ICH S9. The dosing route was oral in 86% of cases for both nonclinical and clinical studies. The majority of nonclinical dosing regimens were daily for 4 weeks (75% of rodent and 74% of non-rodent studies), corresponding to continuous daily clinical dosing (59%). The distribution of compounds that identified a NOAEL, STD10, or HNSTD is summarized in Table 2 along with the number of compounds for which the NOAEL was the same dose or less than the STD10 or HNSTD dose.

**Table 2:** Distribution of NOAEL, STD10, and HNSTD for submitted compounds

	Number of compounds (%)	
	Rodent	Non-rodent
NOAEL determined	55 (60)	63 (68)
STD10 determined	84 (91)	–
HNSTD determined	–	89 (97)
NOAEL = STD10 or HNSTD	10 (11)	22 (24)
NOAEL < STD10 or HNSTD	39 (42)	38 (41)

### Safety of alternative NOAEL-based method and impact on clinical dose escalation

**Fig. 1:**
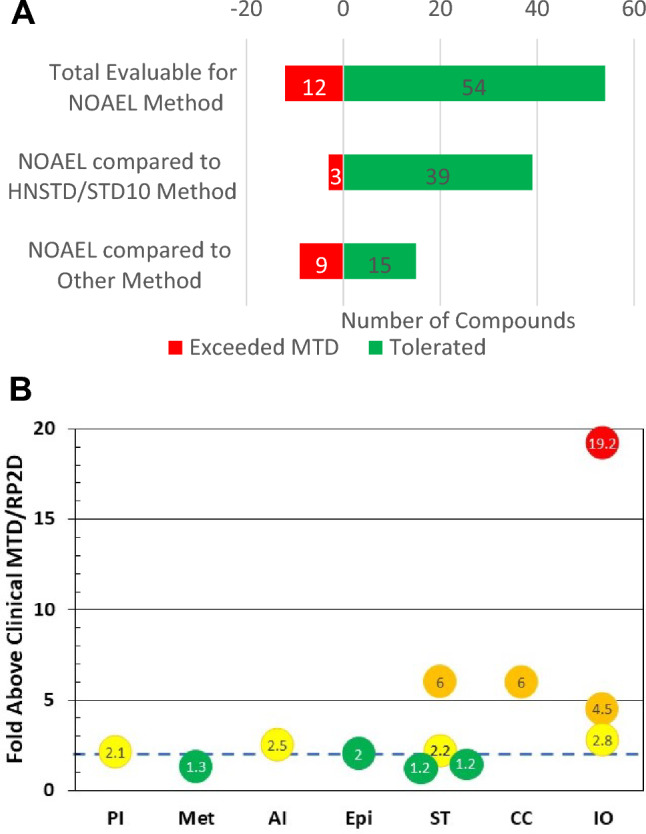
**A** Alternative dose scenario relative to RP2D/MTD. **B** Target class for alternative starting doses exceeding The MTD/RP2D. **A** Number of evaluable cases whose alternative dose would have been tolerated (green) or exceed the RP2D/MTD (red) for all evaluable (top), those that used HNSTD/STD10 (middle), or other starting dose methods (bottom). **B** Fold of the alternative starting dose over the RP2D/MTD by target class (*ST* signal transduction, *PI* proteosome inhibitor, *Met* metabolic, *Epi* epigenetic, *AI* apoptosis inducer, *IO* immuno-oncology, *CC* cell cycle/DNA damage repair) is shown. Green circles are compounds within 2X of clinical MTD/RP2D and also predicted safe, those in yellow possibly safe, while those in orange and red circles may not be safe for initial dose [see text for assessment of these compounds]

Of the 92 compounds, the NOAEL-based evaluation was possible for 66 (72%) compounds resulting in higher alternative starting doses in 60 of these cases (91%), while 6 cases had a lower or similar starting dose than was reported. Of those 66 cases evaluable, 54 (82%) of the NOAEL-based starting doses would have been tolerated in the dose escalation portion of the clinical trial, while 12 (18%) would have exceeded the MTD/RP2D (Fig. 1a) and was independent of species used for the evaluation. For the 60 of 66 cases with higher alternative starting doses, 49 (82%) would not have exceeded the MTD/RP2D. Of the 42 evaluated compounds that originally used HNSTD or STD_10_ to set the starting dose, 39 (93%) would have been tolerated if the NOAEL-based calculation was used, while 15 (63%) of the 24 that used other starting dose methods would have been tolerated using the NOAEL-based method.

The ratio of the alternative NOAEL-based starting dose to the MTD/RP2D revealed that 4 of those compounds that would have exceeded the MTD/RP2D had alternative starting doses that were within twofold of the MTD/RP2D, and an additional 4 compounds were within threefold (Fig. 1b). Three further compounds were between 4.5 to sixfold and 1 molecule was ~ 20-fold the MTD/RP2D. The compound with the highest alternative starting dose relative to the MTD/RP2D and a compound with a 4.5-fold ratio were both immune-oncology (IO) agents delivered by the intra-tumoral (IT) route. Since the IT route is often chosen to limit systemic exposure due to severe tolerability concerns, poor ADME properties, or practical restrictions on the dose volumes from that route, these types of compounds may not be suitable for the NOAEL-based approach and often use the MABEL approach. The only other notable trends with respect to target class for programs where the alternative starting dose exceeded the MTD/RP2D were the absence of cancer mutation and hormone therapy compounds (although these represented small percentages of the total entries), the inclusion of 1 of the 3 total proteasome inhibition compounds, and the inclusion of the only metabolic drug class compound.

A review of the clinical DLTs associated with the cases where the NOAEL-based starting dose exceeded the MTD/RP2D revealed that for the compound with the highest ratio of alternative starting dose to clinical MTD/RP2D, the DLT was “not determined”, indicating that clinical safety may not have been the driver for selection of the RP2D. This may be consistent with the technical restrictions associated with the IT route of administration. Other primary DLTs listed were “liver”, “bone marrow/hematological”, and “systemic toxicity (i.e., systemic inflammation)”, with 2 compounds each, and 1 “cardiovascular-hemodynamic effect.” The remaining primary DLTs were “not determined”, “not disclosed”, or “clinical-other.” Among secondary DLTs, there were 3 “gastrointestinal” and 2 “clinical-emesis.” The nonclinical toxicity of the 12 compounds that would have exceeded the MTD/RP2D showed that none had DLTs that were non-reversible and only 1 had a DLT that was not monitorable.

**Fig. 2:**
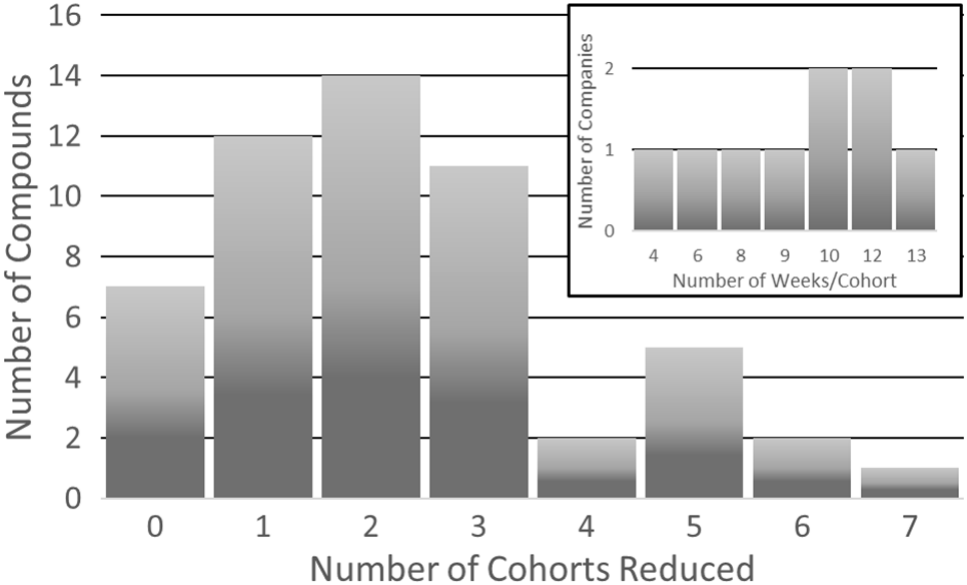
Number of cohorts reduced by alternative method. Among the cases in which the alternative starting dose was less than the RP2D/MTD, the number of fewer cohorts required to reach RP2D/MTD is shown, resulting in a mean of 2.3 and median of 2 cohorts. Inset: the average number of weeks required to complete a dose escalation cohort in an oncology FIP trial was 9.3 weeks (*n* = 9 companies). The average number of weeks reduced using the alternative starting dose method, calculated by multiplying the average number of cohorts reduced (2.3) by the average number of weeks per cohort (9.3), resulting in an average of 21.4 weeks shorter FIP dose escalation period to the reach the RP2D/MTD

Analysis of the entire dataset showed that the average number of clinical cohorts required to reach the MTD/RP2D was 5.3 with a median of 5 cohorts. The average reduction in cohorts using the NOAEL-based alternative starting dose was 2.3 (43%) with a median reduction of 2 cohorts (Fig. 2). The principal factors affecting the time/cohort are presented in Supplemental Fig. 1. As a result, taking the reduced number of cohorts needed to reach the MTD/RP2D using the NOAEL-based approach of 2.3 and the average cohort duration of 9.3 weeks, illustrates a potential time savings of 21.4 weeks in the dose escalation phase.

### Analysis of actual starting dose methods used

As shown in Table 3, the majority of entries in the database used the STD_10_ or HNSTD method of starting dose calculations. As expected, the median safety factors in those cases, 12 × and 8.1 × (highlighted fields), respectively, were similar to the ICH S9/DeGeorge specified safety factors of 10 × and 6 ×, respectively. The calculated number of escalations was reduced using a NOAEL-based method with no safety factor was consistent across all of the groups regardless of which method the sponsor used. In cases where a standard starting dose method (STD_10_ or HNSTD) was used, the application of an alternative NOAEL-based starting dose with no safety factor would have resulted in a tolerated starting dose in > 90% of cases. In contrast, the success of using the alternative NOAEL method with no safety factor was lower (55–75%) in cases where the sponsor used a less common method such as NOAEL (with a safety factor), MABEL, or another alternative. This indicates that additional judgement should be used in determining which method to use; however, in the absence of a reason to be more conservative, a NOAEL method with no safety factor is highly likely to result in a tolerated clinical starting dose and fewer dose escalations prior to reaching RP2D.

**Table 3:** Analysis of sponsor FIP and alternative starting dose methods

Sponsor-reported data					Alternative NOAEL-based starting dose w/o safety factor			
Sponsor method	Instances	Median safety factor		Mean # of escalations^a^	Instances	Starting dose was		Mean # of escalations^a^
		STD10	HNSTD			Tolerated	Not tolerated	
NOAEL	11	97x	33x	4.6	11	6 (55%)	5 (45%)	2.8 (− 39%)
STD_10_	31	12x	13x	5.2	20	19 (95%)	1 (5%)	3.2 (− 38%)
HNSTD	31	18x	8.1x	5.2	22	20 (91%)	2 (9%)	3.8 (− 27%)
MABEL	5	389x	40x	5.6	4	3 (75%)	1 (25%)	3.3 (− 41%)
Alternative	14	39x	23x	5.6	9	6 (67%)	3 (33%)	3.7 (− 34%)

^a^Mean number of dose escalations from starting dose to MTD or RP2D

### Analysis of nonclinical NOAEL

The determination of a NOAEL in toxicology studies conducted to support the clinical dosing of new drugs to oncology patients is not a required endpoint in ICH S9 [2]. However, of the 92 products submitted for the survey, a NOAEL was determined for the majority of rodent (55) and non-rodent (63) toxicology studies (Table 2). In addition, for the purposes of this exercise, companies were able to justify a NOAEL based on the survey criteria (see Methods) for an additional 13 rodent and 11 non-rodent studies. For 22 compounds, a NOAEL was not determined for the rodent and non-rodent studies; of these, 1 justified a rodent NOAEL and 4 justified a non-rodent NOAEL for the alternative human starting dose.

When assessing criteria for justifying the starting dose in clinical studies, it is important to understand species sensitivity to the compound with the more sensitive species most often being used. In preparing to conduct the analysis for using the alternative NOAEL approach, the submitted products were evaluated for how often a NOAEL was determined or justified in both the rodent and non-rodent toxicity studies. Using the criteria outlined for assessing an alternative NOAEL-based starting dose, 66 (72%) of the products were suitable for the exercise.

There were 26 products that did not meet the criteria for the NOAEL-based starting dose assessment (Table 4). Of these 26 products, NOAELs were not identified in both species for 17 products; while 23 rodent studies and 20 non-rodent studies did not report a NOAEL. The target organ toxicity findings that precluded inclusion in the assessment are presented in Table 4. Gastrointestinal, bone marrow or hematologic, renal and skin toxicity were the most common findings for exclusion or absence of a NOAEL determination. For these compounds, the most common observed clinical DLTs were gastrointestinal, bone marrow or hematologic, and skin. It is interesting that cardiac (hemodynamic, electrophysiological, or structural) and hepatic toxicities were not prominent causes given these tissues are often reasons for attrition of non-oncology products [9].

**Table 4:** Primary and secondary DLTs preventing NOAEL determination in animals and patient DLTs for these compounds

DLT	Human (*n* = 26)		Rodent (*n* = 23)		Non-rodent (*n* = 20)	
	Primary	Secondary	Primary	Secondary	Primary	Secondary
Gastrointestinal	3	5	10	4	9	6
Bone marrow or hematologic	6	2	2	6	2	5
Lymphoid	0	0	0	2	1	1
Kidney	1	2	4	0	1	0
Liver	2	1	1	2	1	0
Skin	1	4	0	3	0	1
Pancreas body weight change	2	0	0	0	1	0
Clinical (s)	0	1	2	0	1	1
Cardiovascular	2	0	0	0	0	0
Eye	1	0	1	1	0	0
Bone	0	1	1	1	1	1
Other (biliary, clinical observations [emesis, other], systemic inflammation)	6	2	0	0	2	1
Not determined	2	8	2	4	1	4

### Translation of nonclinical to clinical dose-limiting toxicities

The database collected primary and secondary DLTs from a prespecified lexicon for nonclinical toxicology species and human clinical trials. The lexicon differed slightly between the nonclinical species and clinical trials, which reflected differences in the types of evaluations performed in the studies (e.g., histopathological evaluation in nonclinical studies). The incidence of the DLTs (Table 5) found the predominant rodent and non-rodent dose-limiting toxicities were GI and bone marrow/hematological; GI and bone marrow/hematological dose-limiting toxicities were also most common DLTs in patients. When secondary dose-limiting toxicities were determined, GI and bone marrow/hematological were also among the most prominent across species. Other notable dose-limiting toxicities in humans included skin, liver, and systemic toxicity (i.e., systemic inflammation). In rodent studies, reversibility of dose-limiting toxicities was demonstrated in 58% of cases, while 17% showed partial reversibility, and 10% were predicted to reverse. Only 5% had findings that did not or were predicted not to reverse. Likewise, in non-rodents, 63% of studies showed dose-limiting findings that reversed, 13% partially reversed, and 13% predicted to reverse, leaving only 1 case with irreversible findings. In addition, only 4% of cases demonstrated findings in either rodents or non-rodents deemed unmonitorable.

**Table 5:** Comparison of primary and secondary DLTs across species

DLT	Rodent		Non-rodent		Human	
	Primary	Secondary	Primary	Secondary	Primary	Secondary
Gastrointestinal	29	14	40	9	13	19
Bone marrow/hematologic	14	22	11	20	25	4
Lymphoid	5	6	3	9	0	0
Kidney	8	2	3	0	2	2
Liver	3	6	2	4	7	4
Skin	6	4	1	4	6	10
Clinical (body weight changes)	5	2	3	2	0	1
Clinical (emesis)	–	–	4	0	3	7
Clinical (convulsion)	0	0	2	1	0	0
Clinical (other observations)	3	0	4	1	9	4
Systemic toxicity	1	3	2	3	6	0
Mortality (unknown causes)	3	0	3	0	0	0
Other*	6	10	8	11	11	8
Not determined or NA	9	23	6	28	10	33

*Rodent Other*  bone, pancreas, eye, vasculature, cardiovascular.

*Non-rodent Other*  bone, pancreas, eye, vasculature, cardiovascular (histologic or electrophysiology), clinical (other observations), testes, convulsion, biliary.

*Human Other*  bone, pancreas, eye, cardiovascular (hemodynamic, electrophysiology), CNS, peripheral nerve, testes

## Discussion

The primary goal of selecting a FIP starting dose for cancer patients should be to define a safe dose level that is sufficiently high to potentially result in clinical benefit and to enable rapid identification of the RP2D [9, 10]. A review of 59 approved small molecule oncology MTAs [5] assessed the current approaches to FIP starting dose and dose escalation. While the MTD and RP2D to starting dose ratio was variable, all FIP trials except one had a starting dose lower than the MTD, which indicates that the starting doses have overwhelmingly been safe. A majority of the clinical trials used a 3 + 3 design approach, with a median range of four dose escalation steps required to reach MTD or RP2D from the starting dose. The accelerated titration design, which achieves more rapid dose escalation, was only used in 13% of trials, and the increased use of this approach with single subject cohorts may minimize the number of patients starting clinical trials at sub-therapeutic doses. Twenty nine percent of the FIP trials required ≥ 6 dose escalation steps to reach MTD or RP2D, and there was no difference in the number of dose escalation steps between first-in-class and non-first-in-class molecules.

Similarly, Le Tourneau et al. reviewed the Scopus abstract database for FIP trials of MTAs in cancer patients and concluded the derivation of the starting dose was safe but was based on diverse practices using a variety of nonclinical toxicological parameters (e.g., MTD, TDL, LD10, NOAEL). This analysis showed that starting doses for MTAs were based on rodent and non-rodent data in similar proportions, and overall, starting doses selected using a toxicity-based method did not exceed the human MTD in 96% of clinical trials reviewed. A median of five dose levels was required from the starting dose to the clinical MTD, which is a similar number of cohorts reported by Mittapalli [5]. Our data were consistent with these findings with only 1 actual starting dose exceeding the MTD (99% tolerated), and an average of 5.3 escalations to reach MTD/RP2D (data not shown).

The analysis performed in this paper provides an opportunity to review the optimal starting dose algorithm(s) to enable a reduction in the number of cancer patients starting clinical trials at sub-optimal therapeutic doses and to minimize the number of dose escalation steps and time needed to reach MTD or RP2D. An analysis of 92 small molecule oncology compounds from 12 pharmaceutical companies showed that in more than half of cases the approach used was more conservative than the ICH S9 guideline dictates. In some cases, a less aggressive starting dose was justified based on the data. However, it was rare for starting doses to be significantly more aggressive than the guideline.

**Fig. 3:**
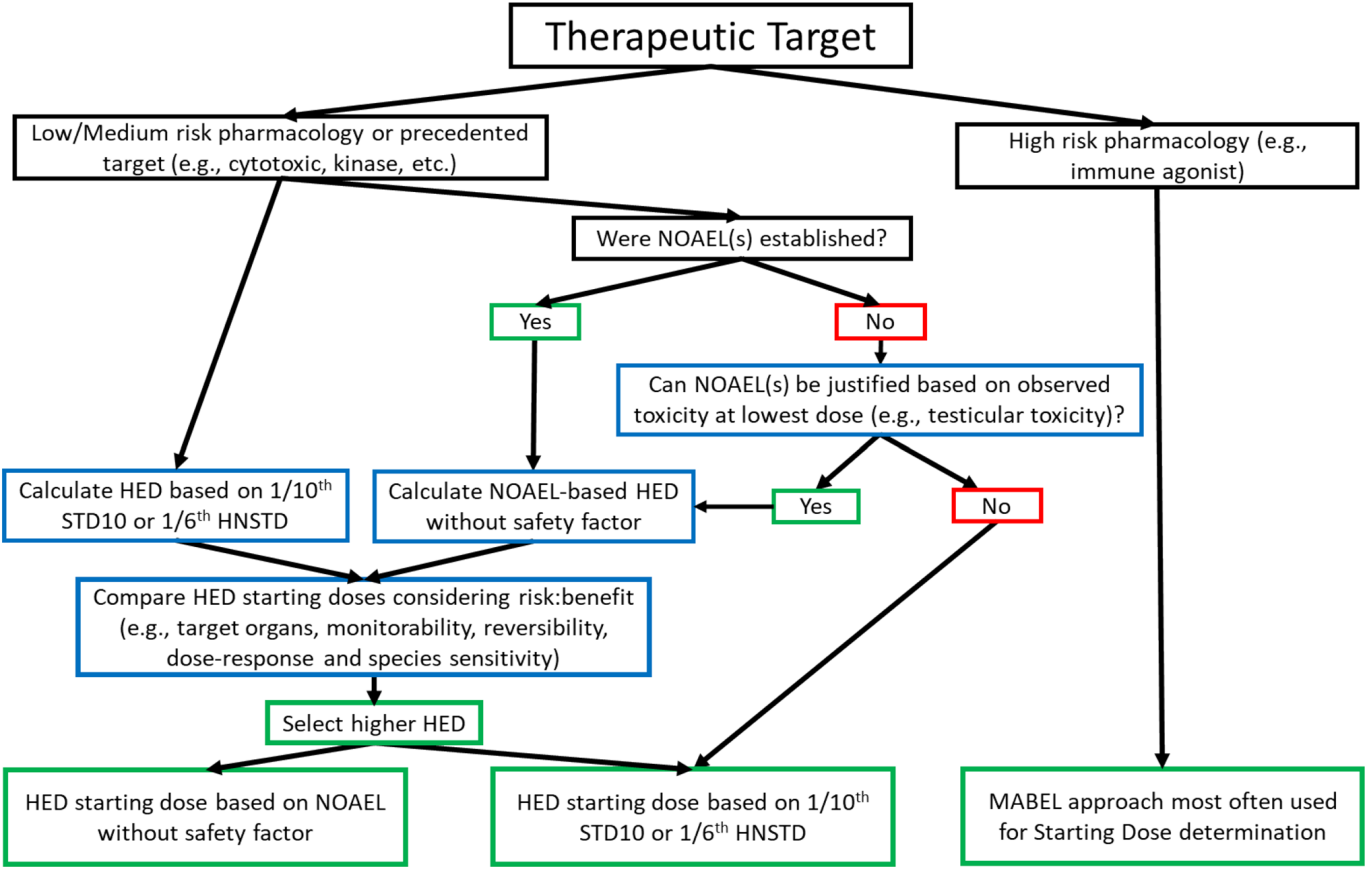
Proposed decision tree for justifying starting dose for FIP oncology studies

Our analysis demonstrates that a less conservative approach to starting dose selection, such as basing the FIP dose on the nonclinical NOAEL without a safety factor, would generally be safe and would reduce the number of dose levels tested in the clinic. To encourage sponsors to be more aggressive in starting dose selection for small molecule oncology therapeutics, thereby reducing the number of patients treated at sub-efficacious dose levels and accelerating drugs to approval, we encourage sponsors to propose and discuss with regulatory agencies using this alternative NOAEL-based method to justify higher starting doses when the data supports it as outlined in Fig. 3. This approach is reflected in the 2019 FDA nonclinical guidance document on severely debilitating or life-threatening hematologic disorders [8]. Although oncology products are not within the scope of the document, this guidance has the similar goal of avoiding administration of sub-therapeutic doses to patients while still protecting patients’ safety. It is worth noting that specifying the NOAEL in toxicology study reports (which may not be common practice for oncology drug candidates) may facilitate the determination of the NOAEL-based alternative starting dose. Of note, as exemplified by the exercise, it may be possible to scientifically propose a NOAEL-based starting dose in the absence of one being identified based on the nature of the finding in the GLP toxicology study and the safety implication of the finding for oncology patients. If sponsors adjust their dose selection strategies for GLP toxicology studies to ensure determination of a NOAEL, this could lead to more broadly spaced dose levels being evaluated and, thus, using the alternative starting dose approach rationale could yield a lower starting dose. In addition, some sponsors may add dose groups to ensure a NOAEL which would not be aligned a 3Rs objective of ICH S9.

In the majority of cases evaluated (82%), the NOAEL-based alternative starting dose would have been safe. The number increases to 93% when evaluating those that originally used the HNSTD or STD10 approach. The reasons for not using the HNSTD or STD10 approach vary, but one common alternative is the use of MABEL. The MABEL is typically applied to immune-oncology agents, mostly those with agonist activity, for which the pharmacological activity is of great safety concern and the translation from nonclinical to clinical safety has lower confidence [10]. Other reasons for using non-traditional approaches include cases where there is low confidence in the translation of predicted exposure or sensitivity of toxicity between animals and humans. The lack of cross-species target potency may also add to the low confidence in translation to humans for some programs. Of the compounds in the database, 60 reported the pharmacologic activity in the rodent toxicology species as unknown and 64 reported the pharmacologic activity in non-rodent toxicology species as unknown. The NOAEL-based alternative starting dose may not be appropriate for cases with increased concern like those discussed above.

Of the 12 (18%) cases where the NOAEL-based alternative starting dose would have exceeded the MTD/RP2D, nonclinical toxicities would have been monitorable in all but one instance. Of these 12 cases, only 3 used HNSTD or STD10 to select the actual starting dose. Others used MABEL, NOAEL with safety factors, or pharmacokinetic/pharmacodynamic modeling. Of the 12 cases, 8 NOAEL-based alternative starting doses were within two–threefold of the clinical MTD/RP2D. Those within twofold of the RP2D would not likely have resulted in significant toxicity as this is within a typical single cohort escalation. Those within threefold might be a slightly more, but possibly acceptable, risk of causing toxicity. Of the remaining 4 cases with NOAEL-based starting doses between 4.5- and 20-fold the MTD/RP2D, 2 were IO agents using IT delivery, one of which did not determine the clinical MTD (implying RP2D was not based on tolerability). IT delivery is often limited by the maximum feasible dose determined by formulation or delivery volume restrictions. In the other 2 cases, both with a NOAEL-based alternative starting dose sixfold the MTD/RP2D, 1 used a NOAEL-based approach with a 50-fold safety factor, implying heightened safety concern, while the other used a LOAEL-based approach with a sixfold safety factor. It should be noted that in the latter case, the actual starting dose was not only the same as the NOAEL-based alternative but was not tolerated and the RP2D was 1/5 the starting dose. Therefore, these 4 cases would not be likely candidates for the NOAEL-based alternative method.

Extensive analysis of translation from nonclinical toxicology studies to clinical findings have been previously published [11, 12]. In general, in our dataset, similar DLTs were observed across toxicology species and humans, with the gastrointestinal system and bone marrow/hematological being the most prominent. In addition, most of the DLTs were reversible or deemed reversible with only 4 cases in rodents and only 1 case in non-rodents that were irreversible (as reported by the company). Additionally, in 78 (85%) of the cases the findings in both rodent and non-rodent were reported as monitorable. In cases without DLTs reported, monitorability was reported as not applicable accounting for differences between 4% of cases demonstrated findings in either rodents or non-rodents deemed unmonitorable and those that were reported to be monitorable.

The decision tree (Fig. 3) outlines points to consider highlighted in this manuscript when assessing the various approaches for selecting the FIP starting dose. Companies should review all possible starting dose calculations and consider the risk–benefit when finalizing the starting dose proposal. When considering the MABEL approach, it is recommended to follow the decision tree in Leach et al. [10]. Another factor to consider in selection of the starting dose is the projected human exposure. The projected human exposure at the clinical starting dose in general should not exceed the exposure in animal toxicity studies at the NOAEL for which potential toxicities of concern were observed. Putting the analyses performed in this paper into perspective of the risk–benefit assessment, the potential benefit of using alternative starting dose methods compared to the risk to exceed the MTD/RP2D using that approach should be taken into consideration. A minimal increase in safety risk may be justified to offer patients with advanced life-threatening disease and limited therapeutic options the potential therapeutic benefit in FIP studies.

In conclusion, retrospective analysis of previously determined starting doses for oncology therapeutics clearly indicate more aggressive starting dose selection should be considered to reduce the use of sub-therapeutic dose levels in oncology patients, and accelerate patient access to effective drugs [1, 13]. These analyses demonstrate that a NOAEL-based alternative starting dose without an added safety factor would have been tolerated in a majority of the cases evaluated, with an anticipated mean reduction of 2.3 cohorts and 21.4 weeks in dose escalation to reach RP2D. The results also indicate a good translation of the absence of adverse effects in animals to the absence of adverse effects in patients, a key message in the Monticello et al. analysis [11]. Alternatively, the rigorous application of rules-based starting dose methods in DeGeorge or ICH S9 could also result in reduced number of cohorts exposed to sub-optimal dose levels with low risk to clinical trial participants. It is unlikely that any method of FIP dose calculation would be appropriate for all oncology small molecule drug candidates and setting the starting dose should be done on a case-by-case approach. However, the NOAEL-based alternative without the application of a safety factor should be calculated, particularly in cases where the HNSTD/STD10 method is typically used and considered if appropriate.

### Supplementary Information

Below is the link to the electronic supplementary material.

Supplementary file1 (XLSX 60 KB)

Supplementary file2 (XLSX 44 KB)

Supplementary file3 (DOCX 54 KB)

## Data Availability

The datasets generated during and/or analyzed during the current study are available from the corresponding author on reasonable request.
