# Correction to: An IQ consortium analysis of starting dose selection for oncology small molecule first‑in‑patient trials suggests an alternative NOAEL‑based method can be safe while reducing time to the recommended phase 2 dose

**DOI:** 10.1007/s00280-023-04595-8

**Published:** 2023-09-29

**Authors:** Bart A. Jessen, Paul Cornwell, Sean Redmond, Thomas Visalli, Marie Lemper, Todd Bunch, Timothy Hart

**Affiliations:** 1 Pfizer, Drug Safety Research and Development, San Diego, CA 92121 USA; 2 Eli Lilly, Nonclinical Safety Assessment, Indianapolis, IN USA; 3 Clinical Pharmacology & Safety Sciences, AstraZeneca Pharmaceuticals, Waltham, MA 02451 USA; 4 Eisai Inc., Global Nonclinical Regulatory, Nutley, NJ 07110 USA; 5 Development Science, UCB, Inc., Cambridge, MA 02140 USA; 6 Nonclinical Safety Evaluation, Bristol Myers Squibb, Princeton, NJ 08540 USA; 7 GlaxoSmithKline, IVIVT, Collegeville, PA 19426 USA

**Correction to: Cancer Chemotherapy and Pharmacology** 10.1007/s00280-023-04570-3

In the original publication, the Fig. 1b has been published incorrectly. The corrected Fig. 1b is given below:
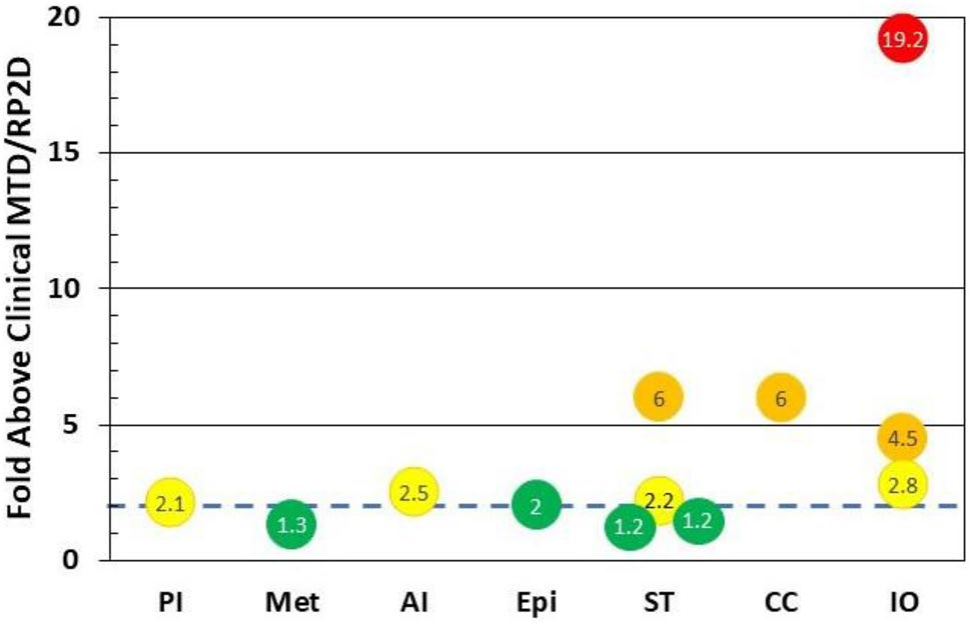


The original article has been corrected.

