# Supplementary material for: An IQ consortium analysis of starting dose selection for oncology small molecule first-in-patient trials suggests an alternative NOAEL-based method can be safe while reducing time to the recommended phase 2 dose
Source: Cancer Chemother Pharmacol. 2023 Jul 28;92(6):455–64. doi: 10.1007/s00280-023-04570-3 (PMC10638197; doi:10.1007/s00280-023-04570-3)
Supplement: Supplementary file 3 — Supplementary file3 (DOCX 54 KB) [file 280_2023_4570_MOESM3_ESM.docx]

**Supplemental Figure 1**

The principal factors affecting the time/cohort identified by companies responding to the supplemental survey (n=9). Responders were asked to select all factors that routinely affect the duration of early dose-escalation cohorts.
